# Outcome in Dilated Cardiomyopathy Related to the Extent, Location, and Pattern of Late Gadolinium Enhancement

**DOI:** 10.1016/j.jcmg.2018.07.015

**Published:** 2019-08

**Authors:** Brian P. Halliday, A. John Baksi, Ankur Gulati, Aamir Ali, Simon Newsome, Cemil Izgi, Monika Arzanauskaite, Amrit Lota, Upasana Tayal, Vassilios S. Vassiliou, John Gregson, Francisco Alpendurada, Michael P. Frenneaux, Stuart A. Cook, John G.F. Cleland, Dudley J. Pennell, Sanjay K. Prasad

**Affiliations:** a Cardiovascular Magnetic Resonance Unit, Royal Brompton Hospital, London; b National Heart & Lung Institute, Imperial College, London; c London School of Hygiene and Tropical Medicine, London; d Norwich Medical School, University of East Anglia, Norwich; e National Heart Centre Singapore, Singapore; f Robertson Centre for Biostatistics, University of Glasgow, Glasgow

**Keywords:** cardiovascular magnetic resonance, dilated cardiomyopathy, late gadolinium enhancement, AIC, Akaike information criterion, CMR, cardiovascular magnetic resonance, DCM, dilated cardiomyopathy, ICD, implantable cardioverter-defibrillators, LGE, late gadolinium enhancement, LVEF, left ventricular ejection fraction

## Abstract

**Objectives:**

This study sought to investigate the association between the extent, location, and pattern of late gadolinium enhancement (LGE) and outcome in a large dilated cardiomyopathy (DCM) cohort.

**Background:**

The relationship between LGE and prognosis in DCM is incompletely understood.

**Methods:**

The authors examined the association between LGE and all-cause mortality and a sudden cardiac death (SCD) composite based on the extent, location, and pattern of LGE in DCM.

**Results:**

Of 874 patients (588 men, median age 52 years) followed for a median of 4.9 years, 300 (34.3%) had nonischemic LGE. Estimated adjusted hazard ratios for patients with an LGE extent of 0 to 2.55%, 2.55% to 5.10%, and >5.10%, respectively, were 1.59 (95% confidence interval [CI]: 0.99 to 2.55), 1.56 (95% CI: 0.96 to 2.54), and 2.31 (95% CI: 1.50 to 3.55) for all-cause mortality, and 2.79 (95% CI: 1.42 to 5.49), 3.86 (95% CI: 2.09 to 7.13), and 4.87 (95% CI: 2.78 to 8.53) for the SCD endpoint. There was a marked nonlinear relationship between LGE extent and outcome such that even small amounts of LGE predicted a substantial increase in risk. The presence of septal LGE was associated with increased mortality, but SCD was most associated with the combined presence of septal and free-wall LGE. Predictive models using LGE presence and location were superior to models based on LGE extent or pattern.

**Conclusions:**

In DCM, the presence of septal LGE is associated with a large increase in the risk of death and SCD events, even when the extent is small. SCD risk is greatest with concomitant septal and free-wall LGE. The incremental value of LGE extent beyond small amounts and LGE pattern is limited.

## Graphical abstract

**Figure:**
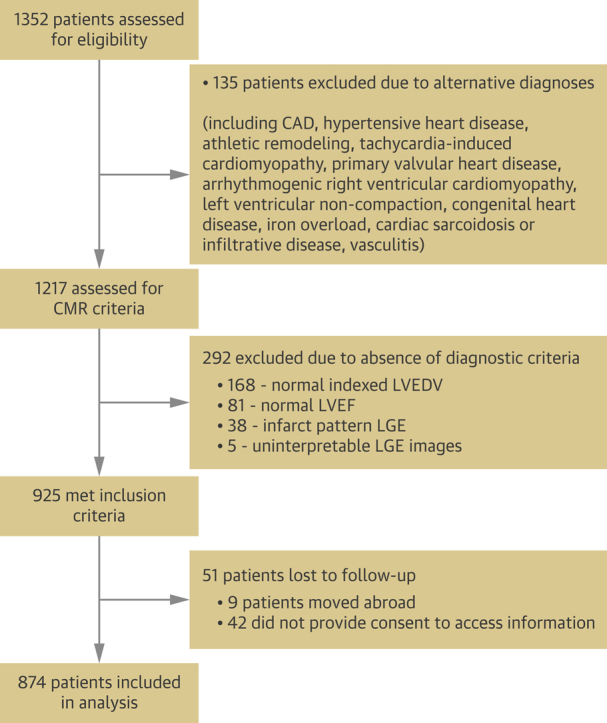

Despite advances in therapy, outcomes in dilated cardiomyopathy (DCM) remain poor (1). DCM is a heterogeneous disease affecting a diverse group of patients and response to therapy is varied (2). Precise phenotyping, enabling targeted and personalized management to improve outcomes and avoid unnecessary interventions remains a therapeutic goal (3).

Late gadolinium enhancement (LGE)-cardiovascular magnetic resonance (CMR) detects nonischemic LGE in approximately 30% of patients, which correlates with replacement fibrosis on histology 1, 4. LGE provides incremental value in addition to left ventricular ejection fraction (LVEF) for predicting all-cause mortality and sudden cardiac death (SCD) events; therefore, it has the potential to guide therapy such as during the selection of patients for implantable cardioverter-defibrillators (ICDs) 1, 4.

Nonischemic LGE most often occurs in a linear pattern in the mid-wall of the septum; however, sub-epicardial patterns and LGE occurring in the free-wall of the left ventricle (LV) are also recognized. The nature of the dose-response relationship between LGE and outcome is poorly understood. Data examining the association between the location and pattern of LGE and specific clinical outcomes are also lacking. Identifying an amount, location, or pattern of LGE that provides the optimal mode of risk stratification will help guide the use of this technique in clinical practice.

## Methods

**Figure 1:**
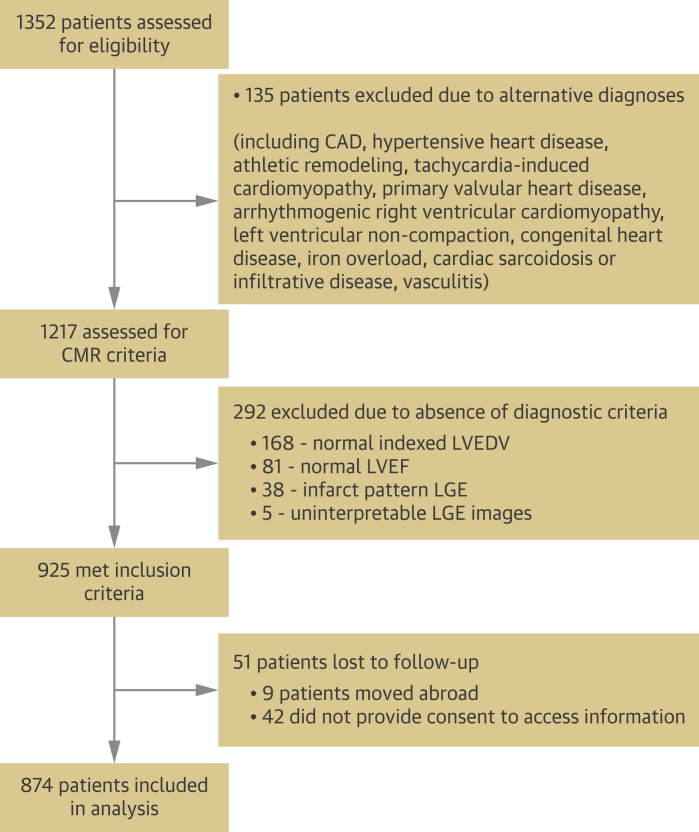
Study Cohort Flow chart detailing the identification of the study cohort.

Consecutive patients with DCM referred to our unit between 2000 and 2011 were screened for a registry. All participants provided written informed consent and the study was approved by the National Research Ethics Service. The diagnosis of DCM was confirmed using the World Health Organization/International Society and Federation of Cardiology definition, based on reduced LVEF and elevated LV end-diastolic volume indexed to body surface area (BSA) (LVEDVi), compared to published age- and sex-specific reference values (5). Exclusion criteria (Figure 1) included ischemic heart disease, defined as a stenosis of >50% in a major coronary artery or evidence of inducible ischemia on functional testing; evidence of acute myocarditis, or ongoing inflammatory myocardial disease; hypertrophic cardiomyopathy; arrhythmogenic right ventricular cardiomyopathy; significant valve disease; and infiltrative disease. In keeping with guidelines 6, 7, an ischemic etiology was considered in all patients and ruled out as follows: All those with infarct patterns of LGE were excluded. Additionally, 681 (77.9%) underwent coronary angiography and 63 (7.2%) perfusion imaging or stress echocardiography without provocation of ischemia. All of the remaining patients (n = 130) were free of angina and considered to have a low risk of ischemic heart disease by their attending cardiologists; the majority (n = 82) were 40 years of age or younger. In the absence of a class 1 indication, coronary angiography was not performed 6, 7. None of these patients underwent coronary revascularization or suffered an acute coronary syndrome during follow-up. The final cohort included 682 from previous studies, all of whom underwent extended follow-up for this study 1, 4.

CMR was performed on a 1.5-T system (Sonata/Avanto, Siemens, Erlangen, Germany) using a standardized protocol (4). Late gadolinium imaging was performed 10 min after intravenous injection of 0.1 mmol/kg of gadopentetate dimeglumine or gadobutrol (Bayer AG, Berlin, Germany) using an inversion-recovery gradient echo sequence. Images were acquired in standard long-axis planes and consecutive short axis slices (8-mm slice thickness with 2-mm gap) in 2-phase encoding directions. Inversion times were optimized to null the myocardium. Ventricular volumes and mass and left atrial volumes were calculated using dedicated software (CMRtools, Cardiovascular Imaging Solutions, London, United Kingdom) and indexed to BSA. The presence of nonischemic LGE was determined by 2 independent operators, with a third providing adjudication if necessary. LGE was considered present if seen in both long- and short-axis planes, in 2 phase-encoding directions, and extending beyond the localized ventricular insertion areas. A senior operator categorized the location and pattern of LGE. The location was classified as septal, LV free-wall, or as occurring in both locations. The pattern was classified as linear mid-wall, sub-epicardial, focal, or as occurring in multiple patterns. LGE quantification was performed by 2 senior operators using the full width at half maximum method (CMR42, Circle Cardiovascular Imaging Inc., Calgary, Canada).

Patients were followed-up from baseline using questionnaires and telephone interviews, and by gathering information from family physicians, cardiologists, and hospital records. Deaths were confirmed using the UK Health and Social Care Information Service. Follow-up time was calculated from the baseline scan until an endpoint occurred or last contact with the patient. All outcome events were adjudicated by a committee of cardiologists blinded to CMR data. The primary outcome of interest was all-cause mortality. The cause of death was confirmed from a combination of medical records, death certification, and postmortem results using American College of Cardiology/American Heart Association guidance (8). The secondary endpoint was an SCD composite including SCD and aborted SCD. SCD was defined as “unexpected death either within 1 h of the onset of cardiac symptoms in the absence of progressive cardiac deterioration; during sleep; or within 24 h of last being seen alive” (9). Aborted SCD was defined as “an appropriate ICD shock for ventricular arrhythmia, successful resuscitation following ventricular fibrillation or spontaneous sustained ventricular tachycardia causing hemodynamic compromise and requiring cardioversion” (8).

### Statistical analysis

To examine the association between LGE extent and outcome, patients with LGE were classified based on the percentage of total myocardial mass occupied by LGE to produce equal tertiles of LGE (>0 and <2.55%, ≥2.55% and <5.10%, and ≥5.10%). Baseline characteristics were compared using the Kruskal-Wallis rank test for continuous data and the Fisher exact test for categorical data. The associations between the extent, location, and pattern of LGE were examined using proportional hazard modelling. Models were adjusted for LVEF, age, and sex given the potential to confound the association between LGE and outcome. As part of a sensitivity analysis, the models were also adjusted for LVEF, age, sex, right ventricular ejection fraction (RVEF), New York Heart Association (NYHA) functional class, LVEDVi, LV mass index, and indexed left atrial volume (LAVi). Results are presented as hazard ratios (HRs) with 95% confidence intervals (CIs). A p value of <0.05 was considered significant. A cubic spline model was fitted to the observed data examining the association between LGE extent and outcome. The cutoff percentage extent of LGE giving the largest C-statistic for the prediction of each end-point was calculated from 1,000 bootstrap samples. The concordance statistic (C-statistic) measured the degree to which a model can distinguish between cases and controls, taking values between 0.5 and 1.0, with larger values indicating better discrimination. The Akaike information criterion (AIC) was used to compare models (10). The AIC allows comparison of nested and non-nested models and reduces the potential of over-fitting the data. Smaller values indicate the optimal model. Interobserver variability in LGE quantification was examined in a random sample of 60 patients with LGE who had quantification performed by both operators, including 20 from each of the group based on extent. The intraclass correlation coefficient was calculated for continuous variables and the Kappa coefficient for categorical variables.

## Results

The final cohort comprised 874 patients, of whom 588 (67.3%) were men, the median LVEF was 39% (interquartile range: 29% to 50%), and nonischemic LGE was present in 300 (34.3%). Baseline characteristics are presented in Table 1. Patients with LGE were older (p = 0.023), more likely to be men (p < 0.0001), and prescribed loop diuretics (p < 0.0001) or mineralocorticoid receptor antagonists (p = 0.008), had lower systolic pressures (p = 0.010) and diastolic blood pressures (p = 0.026), worse NYHA functional class (p = 0.010), lower LVEF (p < 0.0001), and greater LVEDVi (p < 0.0001).

**Table 1:** Baseline Characteristics

	LGE				p Value∗
	No LGE (n = 574)	0.00-2.55% (n = 100)	2.55-5.10% (n = 100)	>5.10% (n = 100)	
Age, yrs	51.0 ± 15.1	52.8 ± 14.4	53.7 ± 14.6	56.2 ± 14.6	0.023
Male	352 (61.3)	80 (80.0)	75 (75.0)	81 (81.0)	<0.0001
BSA, m^2^	1.95 ± 0.24	2.03 ± 0.26	1.97 ± 0.20	1.93 ± 0.21	0.009
Heart rate, beats/min	73.3 ± 13.9	74.9 ± 15.6	73.1 ± 16.0	70.8 ± 14.1	0.26
Systolic blood pressure, mm Hg	121.5 ± 17.6	120.0 ± 16.6	117.8 ± 17.5	115.8 ± 17.3	0.010
Diastolic blood pressure, mm Hg	73.2 ± 11.0	72.2 ± 9.7	71.1 ± 10.5	70.0 ± 11.1	0.026
Atrial fibrillation/flutter	108 (18.8)	23 (23.0)	21 (21.0)	17 (17.0)	0.67
Hypertension	117 (20.4)	25 (25.0)	27 (27.0)	21 (21.0)	0.39
Diabetes	43 (7.5)	17 (17.0)	10 (10.0)	9 (9.0)	0.033
Family history of DCM	52 (9.1)	15 (15.0)	11 (11.1)	8 (8.0)	0.27
Family history of SCD	43 (7.5)	5 (5.0)	7 (7.1)	8 (8.0)	0.85
LBBB	170 (29.7)	29 (29.0)	33 (33.0)	24 (24.2)	0.59
Moderate alcohol excess	64 (11.1)	10 (10.0)	14 (14.0)	12 (12.0)	0.80
Previous chemotherapy	25 (4.4)	6 (6.0)	1 (1.0)	2 (2.0)	0.41
Peripartum diagnosis	14 (2.4)	2 (1.0)	0 (0)	1 (1.0)	0.54
Neuromuscular disease	6 (1.0)	0 (0)	1 (1.0)	1 (1.0)	0.94
Medications					
Beta-blocker	407 (71.0)	76 (76.0)	75 (75.0)	79 (79.0)	0.32
ACE inhibitor	409 (71.3)	73 (73.0)	72 (72.0)	71 (71.0)	0.99
ARB	117 (20.5)	18 (18.0)	21 (21.0)	24 (24.0)	0.76
Loop diuretic	209 (36.4)	63 (63.0)	56 (56.0)	59 (59.0)	<0.0001
Aldosterone antagonist	173 (30.2)	41 (41.0)	43 (43.0)	41 (41.0)	0.008
NYHA functional class					
I	254 (44.4)	33 (33.7)	33 (33.0)	34 (34.3)	0.010
II	229 (40.0)	46 (46.9)	38 (38.0)	41 (41.4)	
III/IV	89 (15.6)	19 (19.4)	29 (29.0)	24 (24.2)	
CMR measurements					
LVEF, %	40.6 ± 12.1	34.4 ± 13.3	35.3 ± 13.1	35.3 ± 12.1	<0.0001
LVEDVi, ml/m^2^	126.3 ± 36.6	147.9 ± 46.1	142.8 ± 49.8	135.5 ± 37.3	<0.0001
LV mass index, g/m^2^	93.0 ± 27.7	108.6 ± 27.0	100.3 ± 24.0	95.7 ± 25.5	<0.0001
RVEF, %	52.4 ± 13.6	48.5 ± 16.5	47.7 ± 15.4	50.6 ± 13.9	0.033
RVEDVi, ml/m^2^	87.9 ± 24.5	94.6 ± 25.8	93.8 ± 30.1	86.4 ± 27.9	0.007
LAVi, ml/m^2^	63.6 ± 25.0	74.3 ± 29.7	69.3 ± 25.8	68.5 ± 27.0	<0.0001

ACE = angiotensin-converting enzyme; ARB = angiotensin II receptor blocker; BSA = body surface area; CMR = cardiovascular magnetic resonance; DCM = dilated cardiomyopathy; LAVi = indexed left atrial volume; LBBB = left bundle branch block; LGE = late gadolinium enhancement; LV = left ventricular; LVEDVi = indexed left ventricular end-diastolic volume; LVEF = left ventricular ejection fraction; RVEDVi = indexed right ventricular end-diastolic volume; RVEF = right ventricular ejection fraction; SCD = sudden cardiac death.

Values are mean ± SD or n (%).

∗ Kruskal-Wallis Rank Test for continuous variables; Fisher Exact Test for categorical variables.

**Figure 2:**
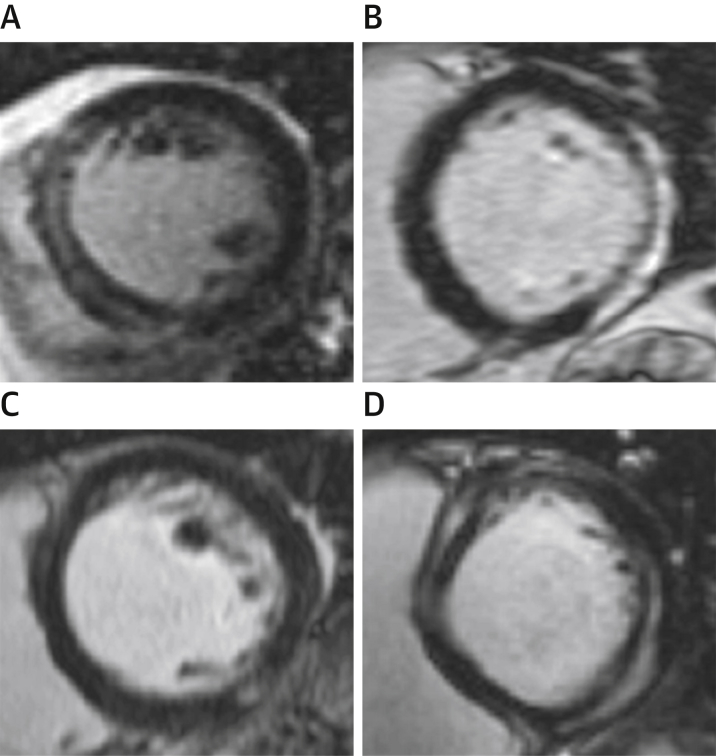
Late Gadolinium Enhancement in Dilated Cardiomyopathy Late gadolinium enhancement images showing **(A)** linear mid-wall enhancement in the septum, **(B)** sub-epicardial enhancement in the lateral wall, **(C)** focal enhancement of the inferior wall, and **(D)** mid-wall enhancement of the septum, lateral and inferior wall.

LGE was present only in the septum in 142 (16.2%) cases, only in the LV free-wall in 42 (4.8%), and in both locations in 116 (13.3%) (Figure 2). LGE was categorized as linear mid-wall in 185 (21.1%) cases, sub-epicardial in 25 (2.9%), focal in 22 (2.5%), and as occurring in multiple patterns in a further 68 (7.8%) (Figure 2).

There was agreement between 2 operators on the presence of LGE in 94.7% of cases (n = 828). There was an absolute mean difference of 0.87% between operators in the quantification of the extent of LGE (intraclass correlation coefficient: 0.87) (Supplemental Figure 1, Supplemental Table 1). Additionally, there was 86.7% agreement in categorizing the LGE extent within 3 groups (>0 and <2.55%, ≥2.55% and <5.10%, and ≥5.10%) (Kappa coefficient: 0.80) (Supplemental Table 2).

### All-cause mortality

Over a median follow-up of 4.9 years (interquartile range: 3.5 to 7.0 years), 150 patients (17.2%) died including 77 (25.7%) with LGE and 73 (12.7%) without (HR: 2.39; 95% CI: 1.73 to 3.29; p < 0.001) (Supplemental Figure 2A). Following adjustment for LVEF, age, and sex, LGE was associated with greater all-cause mortality (HR: 1.81; 95% CI: 1.30 to 2.52; p < 0.001) (Supplemental Table 3). The estimated HRs were similar after additionally adjusting for RVEF, NYHA class, LVEDVi, LV mass index, and LAVi as part of a sensitivity analysis (Supplemental Tables 3 and 4, Supplemental Figure 3).

#### Extent of LGE

**Figure 3:**
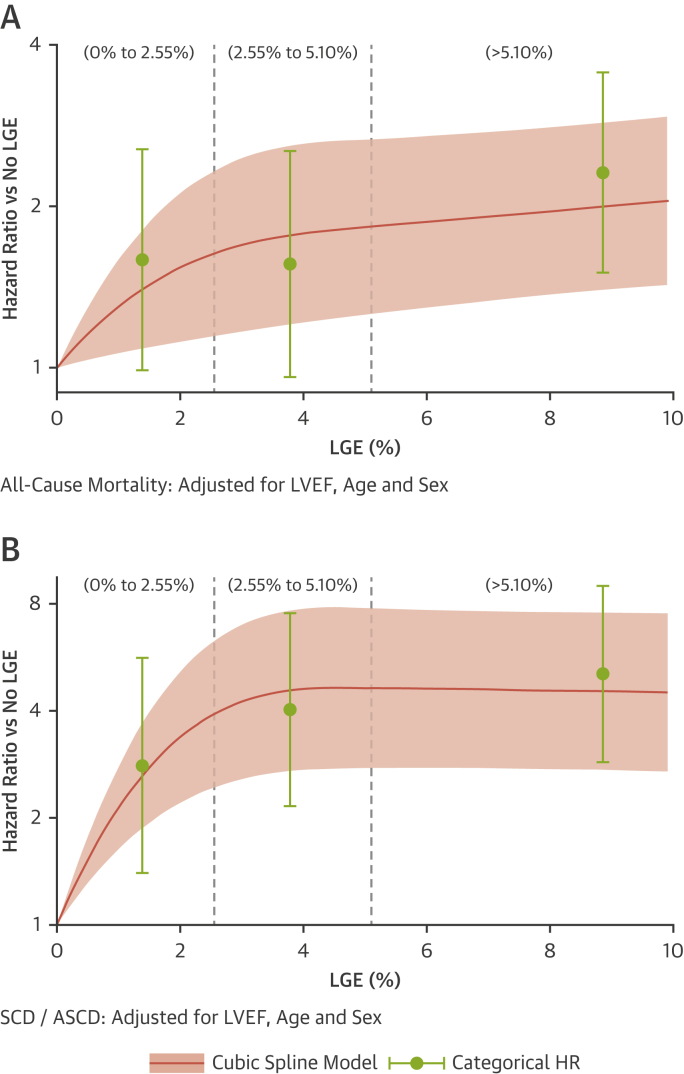
Outcome and Extent of Late Gadolinium Enhancement Estimated adjusted hazard ratios with 95% confidence intervals **(green lines)** for **(A)** all-cause mortality and **(B)** the sudden cardiac death endpoint, per group based on late gadolinium enhancement extent (LGE). Patients are divided into 3 groups based on cut-offs of LGE extent: >0 and <2.55%, ≥2.55 and <5.10%, and ≥5.10%. The hazard ratios for the endpoint are positioned at the median LGE extent within each group. A cubic spline model **(orange line)** has been fitted to the observed data. LVEF = left ventricular ejection fraction; SCD = sudden cardiac death; ASCD = aborted SCD.

**Figure 4:**
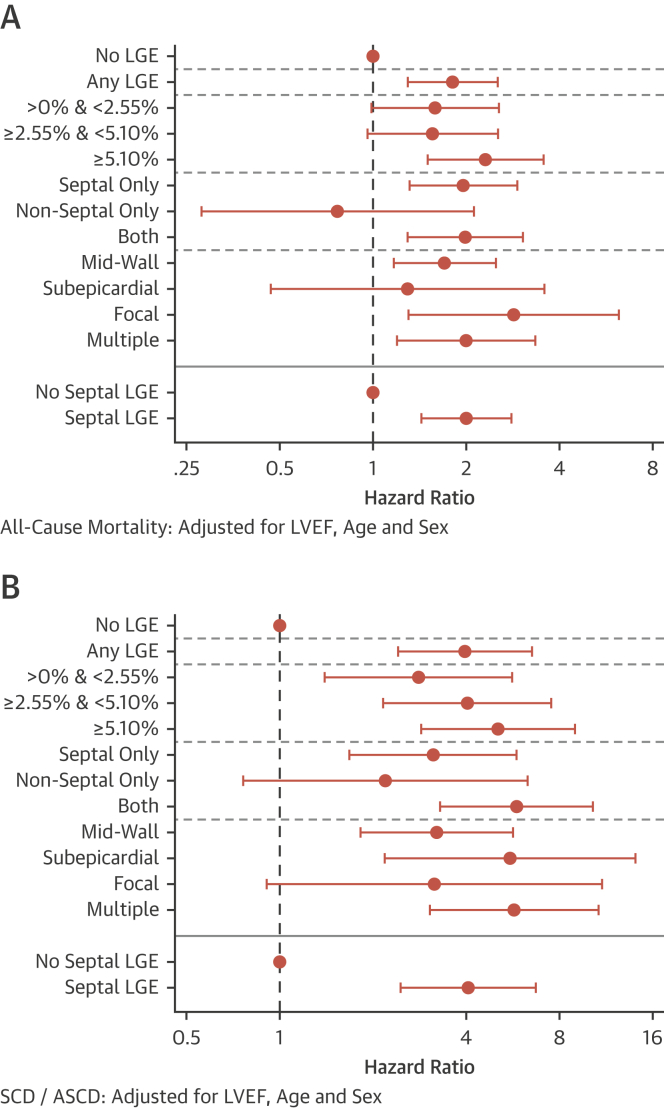
Outcome Related to Extent, Location, and Pattern of Late Gadolinium Enhancement Forrest plots showing the estimated adjusted hazard ratios (HRs) for **(A)** all-cause mortality and **(B)** the sudden cardiac death (SCD) endpoint, per patient group based on late gadolinium enhancement (LGE) extent, location, and pattern. For each model the different LGE HRs are compared to the No-LGE group, except for the final model where “septal LGE” is compared to “no-Septal LGE.” Abbreviations as in Figure 3.

Estimated adjusted HRs for patients with LGE extents of 0 to 2.55%, 2.55% to 5.10%, and >5.10% were 1.59 (95% CI: 0.99 to 2.55; p = 0.056), 1.56 (95% CI: 0.96 to 2.54; p = 0.072), and 2.31 (95% CI: 1.50 to 3.55; p < 0.001), respectively, compared to those without LGE (Figures 3 and 4, Supplemental Figure 2B). Modeling LGE as a linear measure, per percentage increase in extent, underestimated risk in most patients while overestimating risk in the small proportion of patients with the largest extent (Supplemental Figure 4). The percentage extent of LGE giving the largest C-statistic for the primary endpoint was 1.29% (C-statistic: 0.70).

#### Location of LGE

Patients with LGE only in the septum, only in the free-wall, and in both locations had adjusted HRs for the primary endpoint of 1.96 (95% CI: 1.32 to 2.92; p < 0.001), 0.77 (95% CI: 0.28 to 2.12; p = 0.77), and 1.99 (95% CI: 1.30 to 3.04; p = 0.002), compared to those without LGE (Figure 4, Supplemental Figure 2C). A simplified model showed that those patients with septal LGE had an estimated adjusted HR of 2.00 (95% CI: 1.43 to 2.81; p < 0.0001) compared to those without septal LGE (Figure 4).

#### Pattern of LGE

Estimated adjusted HRs for patients with linear mid-wall, sub-epicardial, focal, and multiple patterns of enhancement were 1.70 (95% CI: 1.17 to 2.49; p = 0.006), 1.29 (95% CI: 0.47 to 3.57; p = 0.62), 2.85 (95% CI: 1.30 to 6.23; p = 0.009), and 2.00 (95% CI: 1.20 to 3.34; p = 0.008), respectively, compared to those patients without LGE (Figure 4, Supplemental Figure 2D).

The model with the smallest AIC and the most effective for the prediction of the primary endpoint was based on the presence of septal LGE (Table 2). This was superior to those based on extent or pattern of LGE and the LGE cutoff with the largest C-statistic for the prediction of the primary endpoint. Adding the presence of any LGE and the presence of septal LGE to the baseline multivariable models without LGE improved the C-statistic for the prediction of all-cause mortality (Supplemental Table 3).

**Table 2:** Individual Proportional Hazard Models Investigating the Association Between All-Cause Mortality and Late Gadolinium Enhancement

				Adjusted for LVEF, Sex, and Age				
		n	Mortality	HR (95% CI)	Individual p Value	Overall p Value	C-Statistic	AIC
Presence and extent								
LGE (binary) [any]	0%	574	73 (12.7)	1.00	—	<0.001	0.71	1,790.1
	>0%	300	77 (25.7)	1.81 (1.30 – 2.52)	<0.001			
LGE (binary) [cutoff]	<1.29%	617	81 (13.1)	1.00	—	<0.0001	0.72	1,787.6
	≥1.29%	257	69 (26.8)	1.93 (1.38 – 2.69)	<0.001			
LGE (tertiles)	0%	574	73 (12.7)	1.00	—	0.001	0.72	1,791.5
	>0% and <2.55%	100	24 (24.0)	1.59 (0.99 – 2.55)	0.056			
	≥2.55% and <5.10%	100	22 (22.0)	1.56 (0.96 – 2.54)	0.072			
	≥5.10%	100	31 (31.0)	2.31 (1.50 – 3.55)	<0.001			
Location and pattern								
LGE (by location)	Absent	574	73 (12.7)	1.00	—	<0.001	0.72	1,789.7
	Free-wall only	42	4 (9.5)	0.77 (0.28 – 2.12)	0.61			
	Septal only	142	41 (28.9)	1.96 (1.32 – 2.92)	<0.001			
	Both	116	32 (27.6)	1.99 (1.30 – 3.04)	0.002			
LGE (septal)∗	No	616	77 (12.5)	1.00	—	<0.0001	0.72	1,786.0
	Yes	258	73 (28.3)	2.00 (1.43 – 2.81)	<0.001			
LGE (by pattern)	Absent	574	73 (12.7)	1.00	—	0.005	0.71	1,794.0
	Sub-epicardial	25	4 (16.0)	1.29 (0.47 – 3.57)	0.62			
	Mid-wall	185	47 (25.4)	1.70 (1.17 – 2.49)	0.006			
	Multiple	68	19 (27.9)	2.00 (1.20 – 3.34)	0.008			
	Focal	22	7 (31.8)	2.85 (1.30 – 6.23)	0.009			

Values are n or n (%) unless otherwise indicated.

p values are quoted for each model overall and for the individual components.

AIC = Akaike information criterion; C statistic = Harrell’s C-statistic; CI = confidence intervals; HR = hazard ratio; Pts = number of patients in each sub-group; other abbreviations as in Table 1.

∗ The model with the smallest Akaike information criterion and the most optimal for prediction of all-cause mortality.

### SCD and aborted SCD

Overall, 84 patients (9.6%) suffered SCD or aborted SCD, including 55 patients (18.3%) with LGE and 29 (5.1%) without (HR: 4.12; 95% CI: 2.64 to 6.45; p < 0.001) (Supplemental Figure 5A). Following adjustment for LVEF, age, and sex, LGE was associated with SCD and aborted SCD (HR: 3.96; 95% CI: 2.41 to 6.52; p < 0.001) (Supplemental Table 5). The estimated HRs were similar following adjustment for additional covariates as part of a sensitivity analysis (Supplemental Tables 5 and 6, Supplemental Figure 6).

#### Extent of LGE

Estimated adjusted HRs for patients with LGE extents of 0 to 2.55%, 2.55% to 5.10%, and >5.10%, respectively, were 2.79 (95% CI: 1.42 to 5.49; p = 0.003), 3.86 (95% CI: 2.09 to 7.13; p < 0.0001), and 4.87 (95% CI: 2.78 to 8.53; p < 0.0001), compared to patients without LGE (Figures 3 and 4, Supplemental Figure 5B). Modeling LGE as a linear measure, per percentage increase in extent, underestimated risk in most patients while vastly overestimating risk in the proportion of patients with the largest extent (Supplemental Figure 7). The percentage extent of LGE giving the largest C-statistic for the prediction of the arrhythmic endpoint was 0.71% (C-statistic: 0.70).

#### Location of LGE

Patients with LGE in the septum (HR: 3.13; 95% CI: 1.68 to 5.81; p < 0.001) and in both the septum and free-wall (HR: 5.82; 95% CI: 3.30 to 10.27; p < 0.0001) had greater incidence of the SCD endpoint compared to patients without LGE. Although there was a weaker trend towards increased events in patients with LGE only occurring in the free-wall, this did not reach statistical significance (HR: 2.19; 95% CI: 0.76 to 6.31; p = 0.15) (Figure 4, Supplemental Figure 5C).

#### Pattern of LGE

Estimated adjusted HRs for patients with linear mid-wall, sub-epicardial, focal, and multiple patterns of enhancement were 3.21 (95% CI: 1.82 to 5.66; p < 0.0001), 5.54 (95% CI: 2.18 to 14.08; p < 0.001), 3.16 (95% CI: 0.91 to 10.97; p = 0.070), and 5.72 (95% CI: 3.06 to 10.69; p < 0.0001), respectively, compared to those patients without LGE (Figure 4, Supplemental Figure 5D).

Overall, the model with the smallest AIC that best predicted the SCD endpoint was based on the presence and location of LGE within the septum, the free-wall, or in both locations (Table 3). This was superior to models based on extent and pattern of LGE. Adding the presence of any LGE and the presence of LGE by location to the baseline multivariable models without LGE improved the C-statistic for the prediction of the SCD composite (Supplemental Table 5).

**Table 3:** Individual Proportional Hazard Models Investigating the Association Between Sudden Cardiac Death Events and Late Gadolinium Enhancement

				Adjusted for LVEF, Sex, and Age				
		n	SCD/ASCD	HR (95% CI)	Individual p Value	Overall p Value	C-Statistic	AIC
Presence and extent								
LGE (binary) [any]	0%	574	29 (5.1)	1.00	—	<0.0001	0.70	1027.6
	>0%	300	55 (18.3)	3.96 (2.41 – 6.52)	<0.0001			
LGE (binary) [cutoff]	<1.29%	617	30 (5.2)	1.00	—	<0.0001	0.70	1027.6
	≥1.29%	257	54 (18.6)	3.94 (2.42 – 6.41)	<0.0001			
LGE (tertiles)	0%	574	29 (5.1)	1.00	—	<0.0001	0.71	1028.5
	>0% and <2.55%	100	13 (13.4)	2.80 (1.40 – 5.62)	0.004			
	≥2.55% and <5.10%	100	18 (18.2)	4.03 (2.16 – 7.53)	<0.0001			
	≥5.10%	100	24 (23.1)	5.07 (2.86 – 8.98)	<0.0001			
Location and pattern								
LGE (by location)∗	Absent	574	29 (5.1)	1.00	—	<0.0001	0.72	1024.8
	Free-wall only	42	4 (9.5)	2.19 (0.76 – 6.31)	0.15			
	Septal only	142	21 (14.8)	3.13 (1.68 – 5.81)	<0.001			
	Both	116	30 (25.9)	5.82 (3.30 – 10.27)	<0.0001			
LGE (septal)	No	616	33 (5.4)	1.00	—	<0.0001	0.70	1027.4
	Yes	258	51 (19.8)	4.06 (2.46 – 6.71)	<0.0001			
LGE (by pattern)	Absent	574	29 (5.1)	1.00	—	<0.0001	0.71	1029.5
	Focal	25	3 (13.6)	3.16 (0.91 – 10.97)	0.070			
	Mid-wall	185	29 (15.7)	3.21 (1.82 – 5.66)	<0.0001			
	Sub-epicardial	68	5 (20.0)	5.54 (2.18 – 14.08)	<0.001			
	Multiple	22	18 (26.5)	5.72 (3.06 – 10.69)	<0.0001			

Values are n or n (%) unless otherwise indicated.

p values are quoted for each model overall and for the individual components.

ASCD = aborted sudden cardiac death; all other abbreviations as in Tables 1 and 2.

∗ The model with the smallest AIC and the most optimal for prediction of SCD.

## Discussion

**Figure 5:**
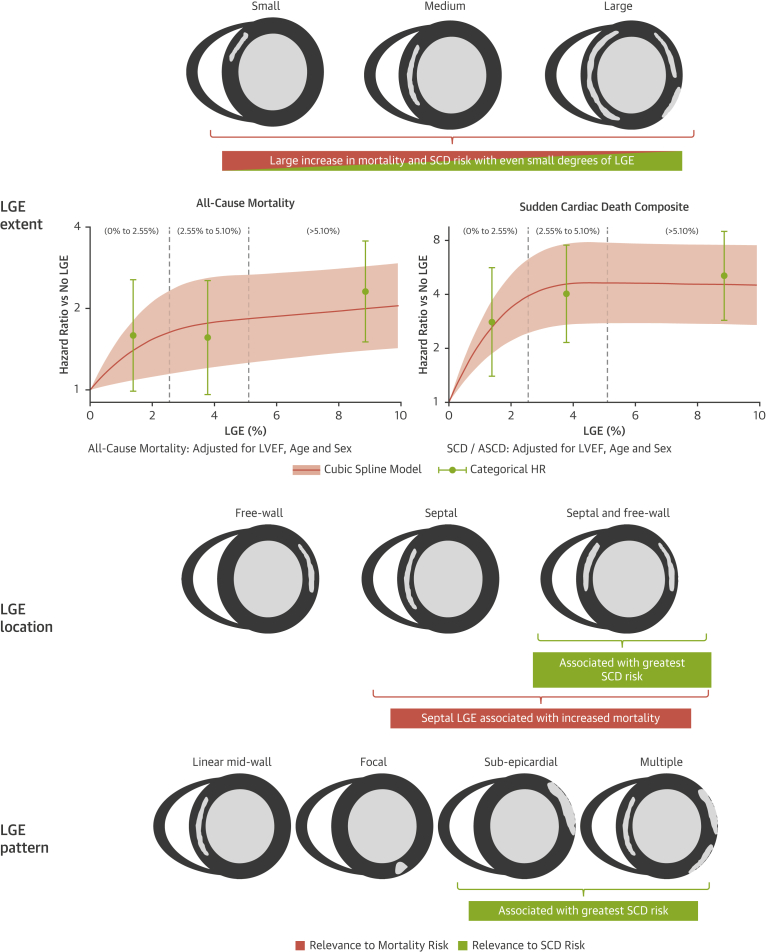
Late Gadolinium Enhancement and Outcome in DCM Our study of dilated cardiomyopathy patients shows a nonlinear relationship between late gadolinium enhancement (LGE) extent and all-cause mortality and sudden cardiac death (SCD) events with a large increase in risk with small degrees of LGE. We show the superiority of models based on the location of LGE for the prediction of these end-points. DCM = dilated cardiomyopathy; other abbreviations as in Figure 3.

This is the largest study to date to examine the association between the extent, location, and pattern of LGE and outcome in a large, well-phenotyped DCM cohort. We show the superiority of models based on the presence and location of LGE for the prediction of all-cause mortality and SCD events over those based on LGE extent and pattern (Figure 5). Our data also establish a nonlinear association between LGE extent and outcome, with a large increase in risk with small degrees of LGE and less marked increases with greater extents thereafter (Figure 5). The increase in risk with small amounts of LGE was most marked for SCD events (Figure 3).

Previous studies have shown that nonischemic LGE is associated with an increased risk of death and arrhythmic events 1, 11. It has been proposed that LGE-CMR may be able to improve the selection of patients who benefit from ICD implantation (12). However, up until now there has been a paucity of data examining the relationship between LGE extent, location, pattern, and specific outcomes.

Our data suggest that measures based on LGE location are better than those based on extent for risk prediction. We show that patients with septal LGE were at highest risk of death whereas those with free-wall LGE were at similar risk to those without LGE. Accordingly, a model based on the presence of septal LGE best predicted all-cause mortality. Whereas septal LGE was also associated with increased SCD events, the greatest risk was seen with concomitant septal and free-wall LGE. A model accounting for the greater risk associated with concomitant LGE in the septum and free-wall was most effective for SCD. Additionally, sub-epicardial or multiple patterns of LGE were associated with a high-risk of SCD events. These data add important new information on how to best to use LGE-CMR in risk stratification, an area of unmet need 12, 13.

Similar to our results, septal LGE has been associated with worse prognosis in myocarditis (14). The variation in risk based on location may be explained by differences in etiological substrate, scar microstructure, and geographical effects. Idiopathic DCM is most commonly associated with septal mid-wall LGE whereas a previous episode of myocarditis, the cause of a third of DCM, is often associated with free-wall LGE (15). Different insults may create fibrosis with different microstructures and varying levels of risk. Septal LGE also has greater interaction with the right ventricle and the conduction system.

Inherited cardiomyopathies may have contributed to the increased risk of SCD events associated with sub-epicardial or multiple patterns of LGE and concomitant LGE in the septum and free-wall. For example, lamin cardiomyopathies are characterized by mid-wall and sub-epicardial LGE in multiple locations and are associated with malignant arrhythmias 16, 17. It is recognized that LV forms of arrhythmogenic cardiomyopathy constitute part of the DCM spectrum (16). Although cases of suspected arrhythmogenic right ventricular cardiomyopathy were excluded, it is possible that our cohort included left-dominant disease, characterized by sub-epicardial fibrofatty replacement. This reflects “real-world” clinical populations. Genetic substrate and fatty infiltration are likely to predispose to arrhythmias in this group (18).

We also show a nonlinear relationship between LGE extent and outcome, such that small degrees of fibrosis are associated with a large increase in risk, particularly with regards to SCD events. This may be explained by the multifactorial disease process. Replacement fibrosis is 1 of several processes contributing to ventricular arrhythmogenesis (3). It is likely that the synergistic presence of multiple features leads to ventricular arrhythmia rather than 1 factor in a linear dose-dependent manner. In addition, it appears that risk is influenced by fibrosis microstructure and heterogeneity, not simply mass. Areas of scar with the greatest heterogeneity will cause the largest variation in conduction velocities and the greatest chance of creating re-entrant arrhythmia. Computational scar modeling offers the potential to provide important insights (19).

Localized LGE at the ventricular insertion areas is common, even in healthy volunteers. What this represents and its significance is uncertain. Examining this was beyond the scope of this study; therefore, localized LGE at the ventricular insertion areas was not included. Quantifying the “gray-zone” surrounding an area of replacement fibrosis was proposed in the context of myocardial infarction (20). There is a lack of histologic correlation examining this concept in DCM. Given the ambiguity over what this technique measures in DCM, we chose not to include it in our analysis.

### Study limitations

Single-center studies are susceptible to selection bias. However, our registry includes patients with a complete spectrum of disease severity referred from secondary and tertiary centers for a range of common indications. In addition, the baseline characteristics are similar to other studies (2). Although data from a proportion of patients have been presented in previous studies 1, 4, patients in this larger cohort had extended follow-up for this investigation. The large number of patients and events affords greater statistical power and enables the investigation of multiple statistical models. The smaller number of patients in sub-groups such as those with focal or sub-epicardial LGE does, however, limit the interpretation of this specific data.

We recognize that not all arrhythmias resulting in appropriate shocks may have resulted in SCD if untreated. However, we have selected the most robust definition available, excluding antitachycardia pacing (8). We acknowledge that the use of different contrast agents has the potential to impact LGE quantification. However, there was no difference in the quantity, pattern, or location of LGE for patients scanned with gadobutrol compared to gadopentetate dimeglumine. In addition, the associations between LGE and outcome remain similar when patients are divided based on contrast agent and there was no difference in the estimated effect of LGE on outcome between groups (Supplemental Table 7). The impact of the use of different contrast agents on the results of the study, therefore, appears to be minimal.

Parametric mapping was not available at the outset of the current study and was therefore not included in the analysis. This technique has the advantage of identifying diffuse myocardial changes which LGE imaging may not detect. Previous work has shown associations between native T1 values and mortality and heart failure outcomes in DCM (21). Given the possible role of diffuse fibrosis in arrhythmia generation and heart failure, parametric mapping offers hope in the identification of those at risk of adverse outcomes. We eagerly await further data examining the incremental value of parametric mapping. Our data suggest the need to examine the incremental value of this technique in addition to the presence of septal LGE.

## Conclusions

We show a large increase in all-cause mortality and SCD risk with small amounts of LGE. The incremental value of LGE extent is therefore limited. In addition, we show that septal LGE is associated with all-cause mortality and concomitant LGE in the septum and free-wall is associated with the greatest risk of SCD events.

Perspectives **COMPETENCY IN MEDICAL KNOWLEDGE:** The presence of even small degrees of LGE is associated with an increased risk of death and SCD events. The presence of septal LGE is the best marker of mortality risk whilst the presence of concomitant LGE in the septum and free-wall confers the highest risk of SCD events.

**TRANSLATIONAL OUTLOOK:** Randomized trials are needed to investigate whether patients with LGE gain benefit from targeted therapies, such as ICD implantation or novel antifibrotic agents.

## References

[1] Gulati A., Jabbour A., Ismail T.F. (2013). Association of fibrosis with mortality and sudden cardiac death in patients with nonischemic dilated cardiomyopathy. JAMA.

[2] McNamara D.M., Starling R.C., Cooper L.T. (2011). Clinical and demographic predictors of outcomes in recent onset dilated cardiomyopathy: results of the IMAC-2 study. J Am Coll Cardiol.

[3] Halliday B.P., Cleland J.G.F., Goldberger J.J., Prasad S.K. (2017). Personalizing risk stratification for sudden death in dilated cardiomyopathy: the past, present, and future. Circulation.

[4] Halliday B.P., Gulati A., Ali A. (2017). Association between midwall late gadolinium enhancement and sudden cardiac death in patients with dilated cardiomyopathy and mild and moderate left ventricular systolic dysfunction. Circulation.

[5] Maceira A.M., Prasad S.K., Khan M., Pennell D.J. (2006). Normalized left ventricular systolic and diastolic function by steady state free precession cardiovascular magnetic resonance. J Cardiovasc Magn Reson.

[6] Yancy C.W., Jessup M., Bozkurt B. (2013). 2013 ACCF/AHA guideline for the management of heart failure. Circulation.

[7] Ponikowski P., Voors A.A., Anker S.D. (2016). 2016 esc guidelines for the diagnosis and treatment of acute and chronic heart failure. Eur Heart J.

[8] Buxton A.E., Calkins H., Callans D.J. (2006). ACC/AHA/HRS 2006 key data elements and definitions for electrophysiological studies and procedures. J Am Coll Cardiol.

[9] Hicks K.A., Tcheng J.E., Bozkurt B. (2015). 2014 ACC/AHA key data elements and definitions for cardiovascular endpoint events in clinical trials. Circulation.

[10] May M., Royston P., Egger M., Justice A.C., Sterne J.A., Collaboration A.R.T.C. (2004). Development and validation of a prognostic model for survival time data: application to prognosis of HIV positive patients treated with antiretroviral therapy. Stat Med.

[11] Disertori M., Rigoni M., Pace N. (2016). Myocardial fibrosis assessment by LGE is a powerful predictor of ventricular tachyarrhythmias in ischemic and nonischemic LV dysfunction: a meta-analysis. J Am Coll Cardiol Img.

[12] Arbustini E., Disertori M., Narula J. (2017). Primary prevention of sudden arrhythmic death in dilated cardiomyopathy: current guidelines and risk stratification. J Am Coll Cardioil HF.

[13] Kober L., Thune J.J., Nielsen J.C. (2016). Defibrillator implantation in patients with nonischemic systolic heart failure. N Engl J Med.

[14] Grani C., Eichhorn C., Biere L. (2017). Prognostic value of cardiac magnetic resonance tissue characterization in risk stratifying patients with suspected myocarditis. J Am Coll Cardiol.

[15] Mahrholdt H., Wagner A., Deluigi C.C. (2006). Presentation, patterns of myocardial damage, and clinical course of viral myocarditis. Circulation.

[16] Pinto Y.M., Elliott P.M., Arbustini E. (2016). Proposal for a revised definition of dilated cardiomyopathy, hypokinetic non-dilated cardiomyopathy, and its implications for clinical practice. Eur Heart J.

[17] Hasselberg N.E., Haland T.F., Sabernieak J. (2018). Lamin A/C cardiomyopathy: young onset, high penetrance, and frequent need for heart transplantation. Eur Heart J.

[18] Arbustini E., Kramer C.M., Narula J. (2018). Arrhythmogenic potential of border zone after myocardial infarction: scar is more than just a healed wound. J Am Coll Cardiol Img.

[19] Arevalo H.J., Vadakkumpadan F., Guallar E. (2016). Arrhythmia risk stratification of patients after myocardial infarction using personalized heart models. Nat Commun.

[20] Yan A.T., Shayne A.J., Brown K.A. (2006). Characterization of the peri-infarct zone by contrast-enhanced cardiac magnetic resonance imaging is a powerful predictor of post-myocardial infarction mortality. Circulation.

[21] Puntmann V.O., Carr-White G., Jabbour A. (2016). T1-mapping and outcome in nonischemic cardiomyopathy: all-cause mortality and heart failure. J Am Coll Cardiol Img.

