# Supplementary material for: Outcome in Dilated Cardiomyopathy Related to the Extent, Location, and Pattern of Late Gadolinium Enhancement
Source: JACC Cardiovasc Imaging. 2019 Aug;12(8):1645–55. doi: 10.1016/j.jcmg.2018.07.015 (PMC6682609; doi:10.1016/j.jcmg.2018.07.015)
Supplement: Supplemental Figures 1–7 and Supplemental Tables 1–7 [file mmc1.doc]

**Supplementary Appendix**

**Online Figure 1, Interobserver reproducibility**

Caption: Bland-Altmann plot illustrating the difference between two operators in the quantification of late gadolinium enhancement, with the mean of the two measurements on the x-axis and the difference between the two measurements on the y-axis.


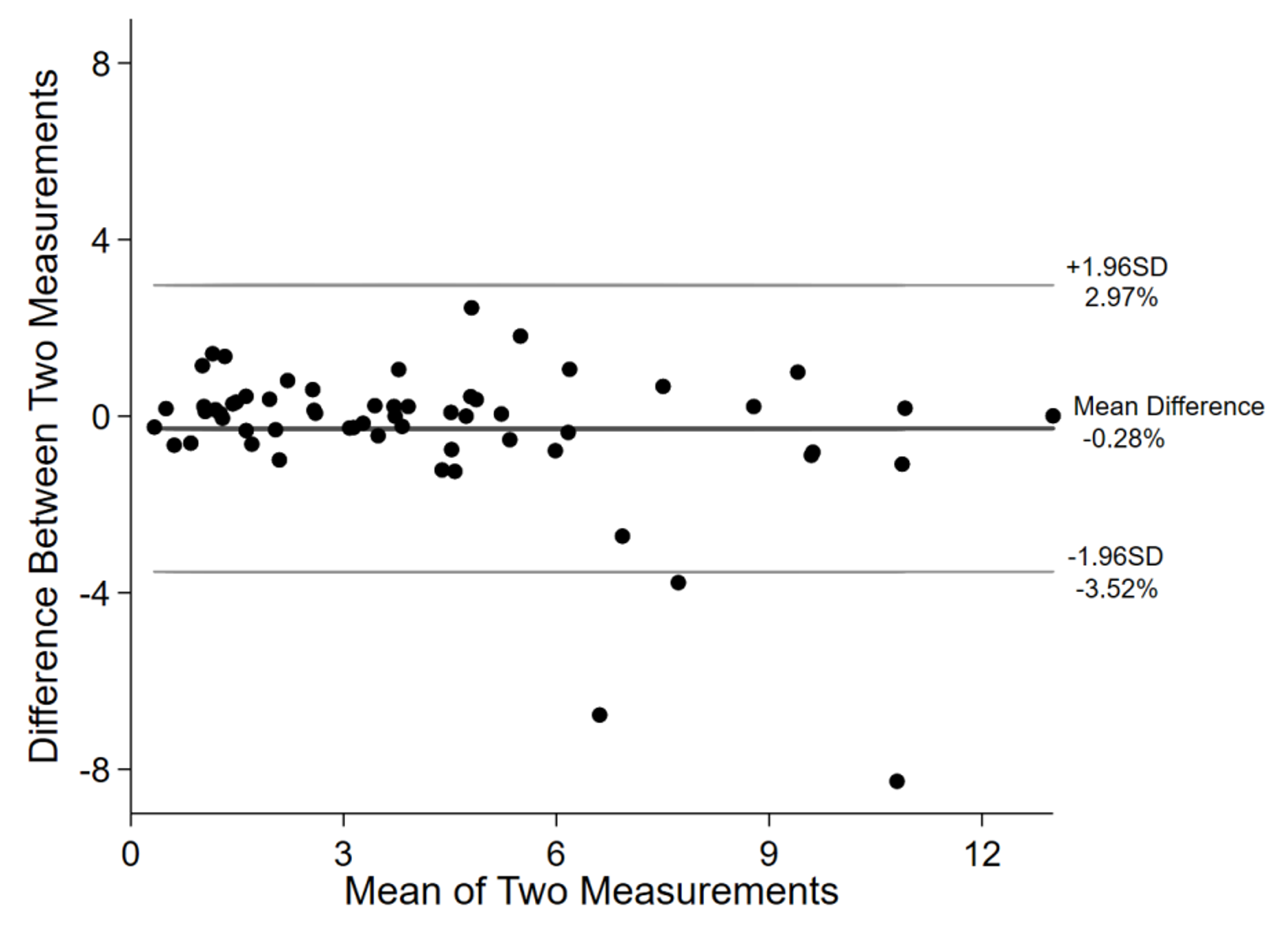


**Online Figure 2. Kaplan Meier Curves examining the association between late gadolinium enhancement and all-cause mortality.**

Caption: Kaplan-Meier curves examining the time to death based on the (A) presence of late gadolinium enhancement (LGE), (B) the extent of LGE, (C) the location of LGE and (D) the pattern of LGE. (LMW – mid-wall)


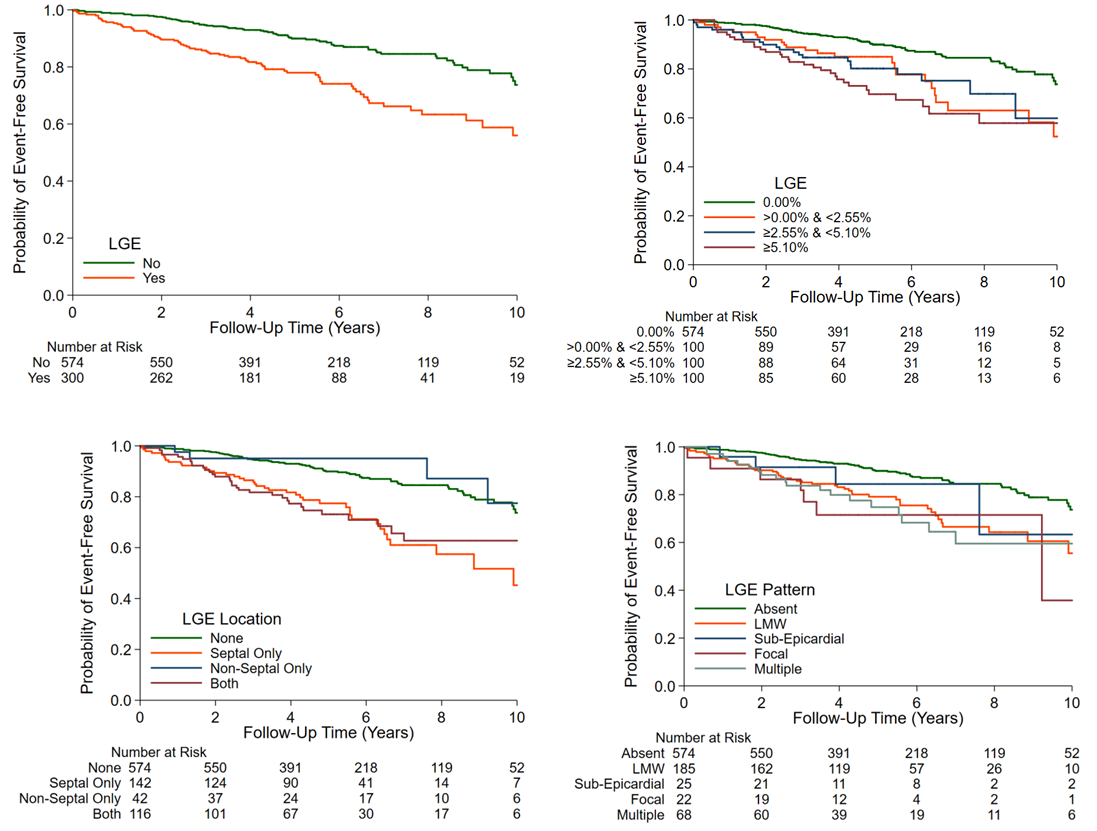


**Online Figure 3. Kaplan-Meier Curves examining the association between the co-variates used in the sensitivity analysis and all-cause mortality**

Caption: Kaplan-Meier curves examining the time to death based on (A) age (LGE), (B) LAVi, (C) LVEDVi, (D) LVEF, (E) LV mass index, (F) NYHA class, (G) RVEF and (H) sex.


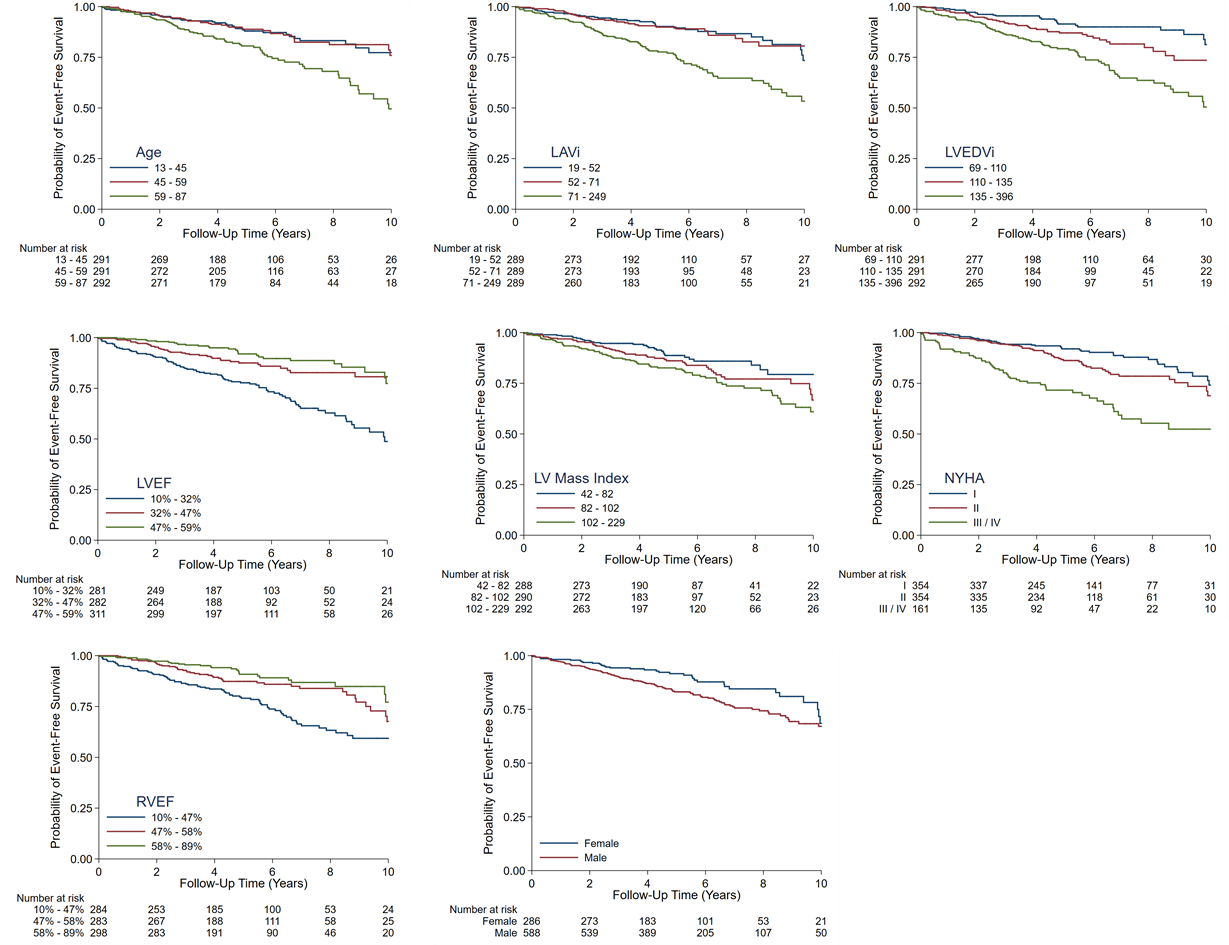


**Online Figure 4. Association between late gadolinium extent and all-cause mortality**

Caption: Association between all-cause mortality and late gadolinium extent. A cubic spline curve has been fitted to the observed data (blue line). The data was also modelled based on a linear relationship, per percent increase in extent (red line), demonstrating under-prediction of risk at small extents and over-prediction of risk at larger extents.


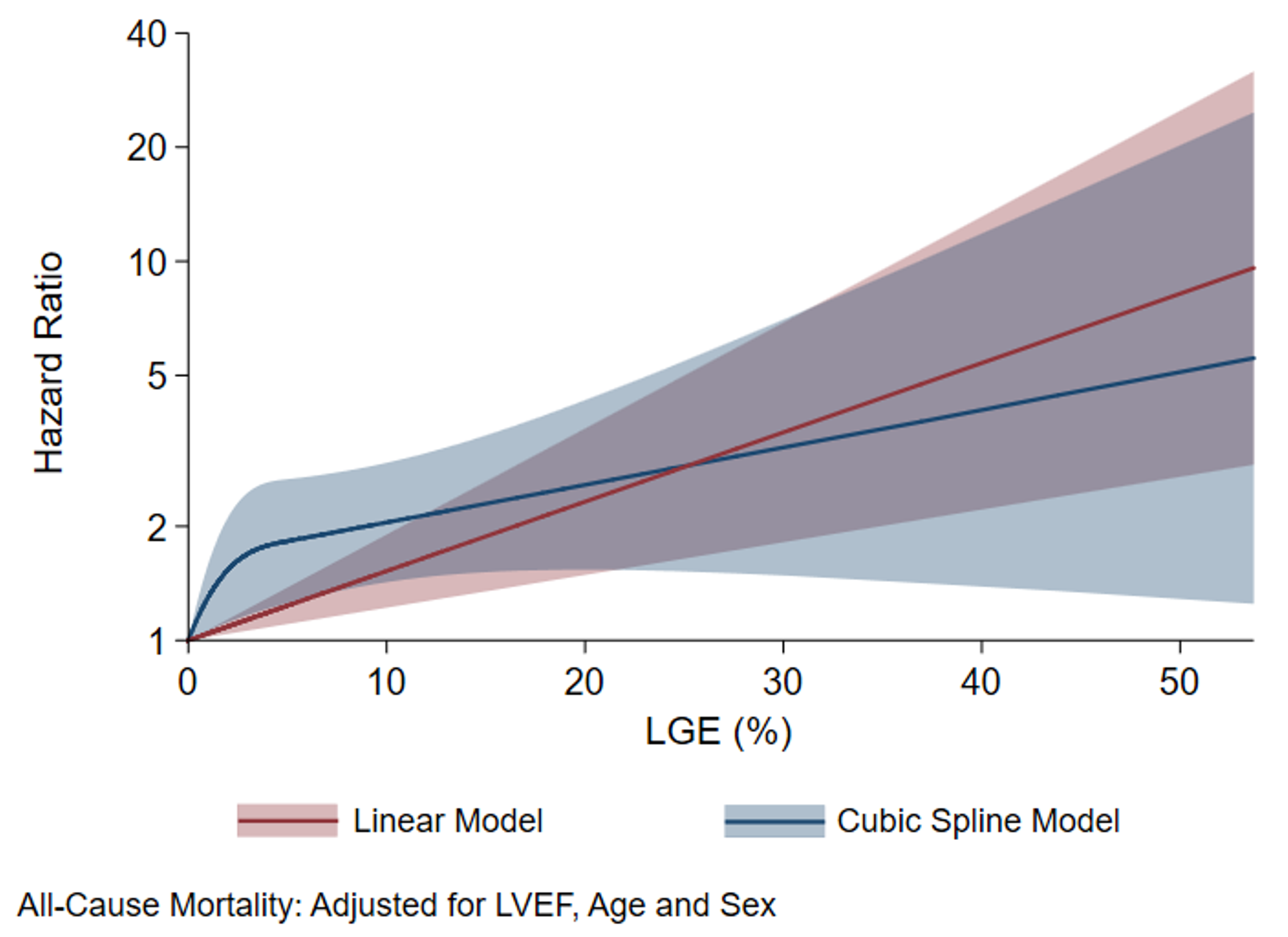


**Online Figure 5. Kaplan Meier Curves examining the association between late gadolinium enhancement and sudden cardiac death events.**

Caption: Kaplan-Meier curves examining the time to the composite sudden death end-point based on the (A) presence of late gadolinium enhancement (LGE), (B) the extent of LGE, (C) the location of LGE and (D) the pattern of LGE. (LMW – mid-wall)


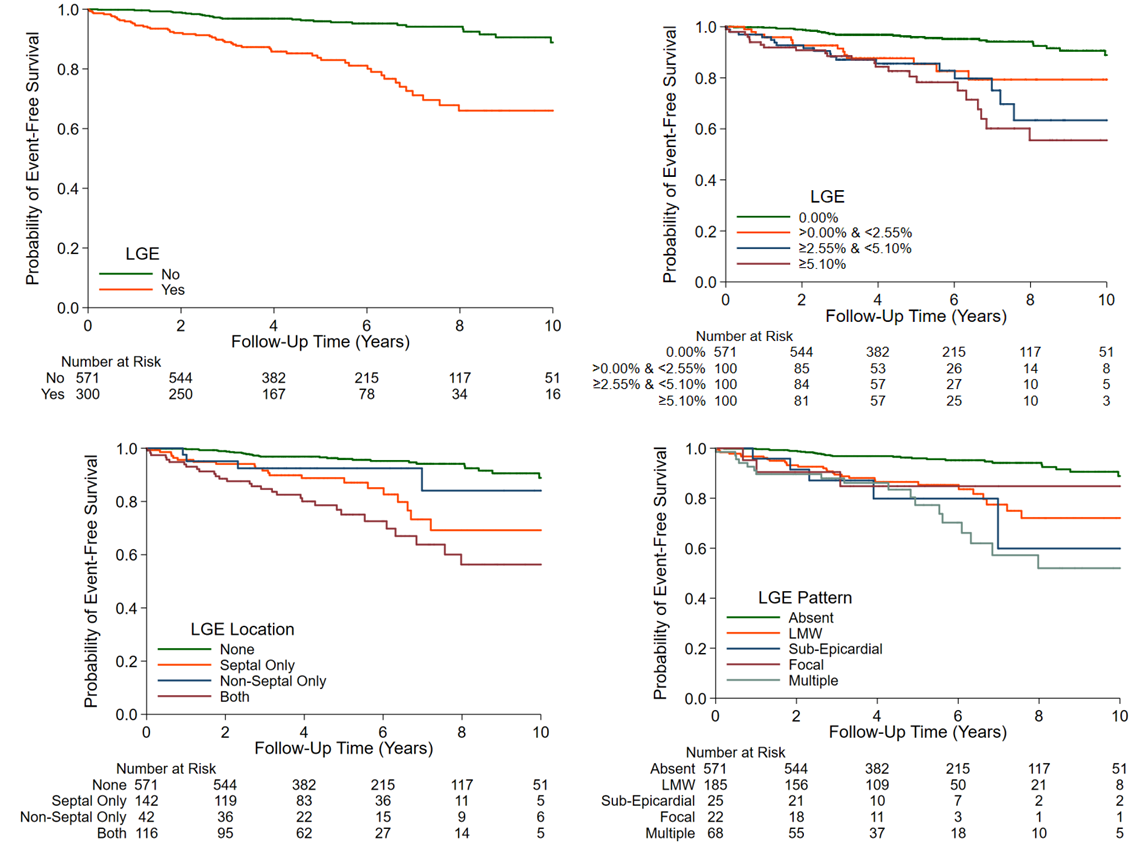


**Online Figure 6. Kaplan-Meier Curves examining the association between the co-variates used in the sensitivity analysis and SCD events**

Caption: Kaplan-Meier curves examining the time to event based on (A) age (LGE), (B) LAVi, (C) LVEDVi, (D) LVEF, (E) LV mass index, (F) NYHA class, (G) RVEF and (H) sex.


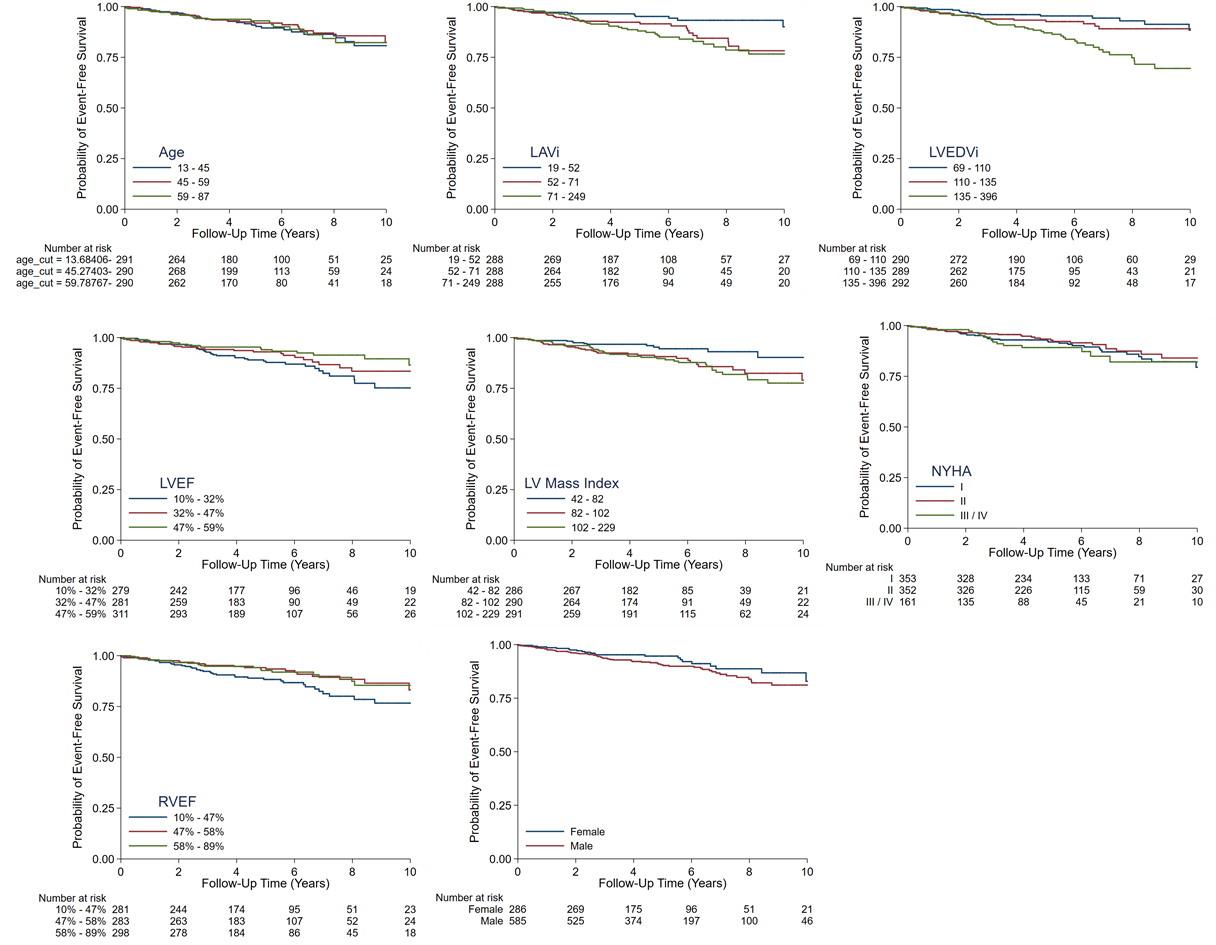


**Online Figure 7. Association between late gadolinium extent and sudden cardiac death events**

Caption: Association between SCD events and late gadolinium extent. A cubic spline curve has been fitted to the observed data (blue line). The data was also modelled based on a linear relationship, per percent increase in extent (red line), demonstrating under-prediction of risk at small extents and over-prediction of risk at larger extents.


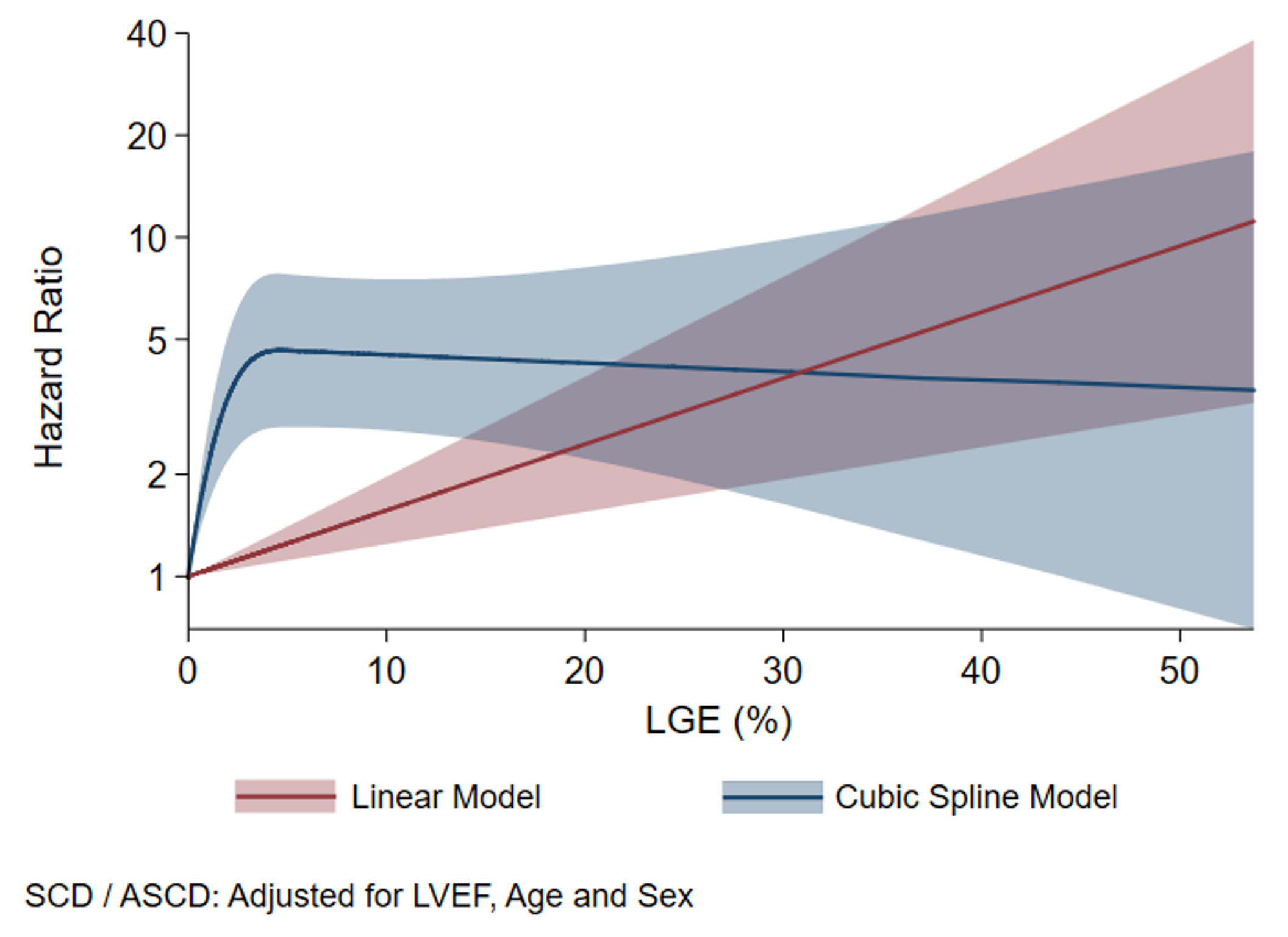


**Online Table 1. Interobserver reproducibility in late gadolinium quantification**

| Observer 1 | Observer 2 | Absolute Mean Difference (SD) | ICC (95% CI) |
| --- | --- | --- | --- |
| Mean (SD) | Mean (SD) |
| 4.10 (2.91) | 4.38 (3.46) | 0.87 (1.43) | 0.87 (0.79, 0.92) |

Mean quantity of late gadolinium enhancement calculated by two operators and the intraclass correlation coefficient

**Online Table 2. Interobserver reproducibility in late gadolinium quantification within three specific categories**

|  |  | Observer 2 | | |
| --- | --- | --- | --- | --- |
|  | Group | 1 | 2 | 3 |
| Observer 1 | 1 | 19 | 1 | 0 |
|  | 2 | 2 | 17 | 2 |
|  | 3 | 0 | 3 | 16 |
|  |  | 86.7% Agreement | Kappa = 0.80 |  |

Classification of the quantity of late gadolinium enhancement within three tertiles by two operators and the Kappa coefficient of variation

**Online Table 3. Univariable and multivariable models for all-cause mortality**

| **Univariable Models (Outcome ACM)** | | |
| --- | --- | --- |
|  | **HR (95% CI)** | **P** |
| LVEF (per 10%) | 0.63 (0.56, 0.71) | <0.0001 |
| LGE (binary) | 2.39 (1.73, 3.29) | <0.0001 |
| NYHA II | 1.49 (0.99, 2.24) | <0.0001 |
| NYHA III / IV | 3.62 (2.40, 5.46) |
| LVEDVi (per 10ml/m^2^) | 1.10 (1.07, 1.13) | <0.0001 |
| RVEF (per 10%) | 0.71 (0.64, 0.79) | <0.0001 |
| LAVi (per 10ml/m^2^) | 1.13 (1.08, 1.19) | <0.0001 |
| Age (per 10 years) | 1.32 (1.17, 1.48) | <0.0001 |
| LVMi (per 10g/m^2^) | 1.10 (1.05, 1.16) | <0.001 |
| Male | 1.60 (1.10, 2.35) | 0.014 |

Multivariable Model 1

| **Multivariable Model 1 without LGE (Outcome ACM)** | | | |
| --- | --- | --- | --- |
|  | HR (95% CI) | P | C-statistic |
| LVEF (per 10%) | 0.64 (0.56, 0.73) | <0.00001 | 0.69 |
| Age (per 10 years) | 1.31 (1.16, 1.47) | <0.00001 |
| Male | 1.52 (1.04, 2.23) | 0.030 |

| **Multivariable Model 1 with LGE (Outcome ACM)** | | | |
| --- | --- | --- | --- |
|  | HR (95% CI) | P | C-statistic |
| LVEF (per 10%) | 0.66 (0.58, 0.76) | <0.00001 | 0.71 |
| Age (per 10 years) | 1.30 (1.16, 1.46) | <0.00001 |
| Any LGE | 1.81 (1.30, 2.52) | <0.001 |
| Male | 1.34 (0.91, 1.98) | 0.14 |

| **Multivariable Model 1 with septal LGE (Outcome ACM)** | | | |
| --- | --- | --- | --- |
|  | HR (95% CI) | P | C |
| LVEF (per 10%) | 0.68 (0.59, 0.78) | <0.00001 | 0.72 |
| Age (per 10 years) | 1.31 (1.16, 1.47) | <0.00001 |
| Septal LGE | 2.00 (1.43, 2.81) | <0.0001 |
| Male | 1.34 (0.91, 1.98) | 0.14 |

Multivariable Model 2

| **Multivariable Model 2 without LGE (All-cause mortality)** | | | |
| --- | --- | --- | --- |
|  | HR (95% CI) | P | C-statistic |
| Age (per 10 years) | 1.37 (1.21, 1.55) | <0.00001 | 0.73 |
| NYHA II | 1.25 (0.82, 1.91) | <0.001 |
| NYHA III / IV | 2.37 (1.46, 3.84) |
| RVEF (per 10%) | 0.83 (0.72, 0.97) | 0.016 |
| LVEDVi (per 10) | 1.06 (1.02, 1.11) | 0.007 |
| Male | 1.73 (1.15, 2.61) | 0.009 |
| LVMi (per 10) | 0.96 (0.90, 1.03) | 0.27 |
| LAVi (per 10) | 1.02 (0.96, 1.09) | 0.43 |
| LVEF (per 10%) | 0.93 (0.75, 1.14) | 0.46 |

| **Multivariable Model 2 with LGE (All-cause mortality)** | | | |
| --- | --- | --- | --- |
|  | HR (95% CI) | P | C-statistic |
| **Any LGE** | **1.70 (1.21, 2.39)** | **0.002** | 0.74 |
| Age (per 10 years) | 1.37 (1.21, 1.55) | <0.00001 |
| NYHA II | 1.21 (0.79, 1.85) | 0.001 |
| NYHA III / IV | 2.26 (1.40, 3.67) |
| RVEF (per 10%) | 0.83 (0.71, 0.96) | 0.012 |
| LVEDVi (per 10) | 1.06 (1.01, 1.11) | 0.014 |
| Male | 1.53 (1.00, 2.33) | 0.049 |
| LVMi (per 10) | 0.96 (0.90, 1.03) | 0.29 |
| LAVi (per 10) | 1.02 (0.96, 1.08) | 0.54 |
| LVEF (per 10%) | 0.94 (0.77, 1.15) | 0.56 |

| **Multivariable Model 2 with septal LGE (Outcome ACM)** | | | |
| --- | --- | --- | --- |
|  | **HR (95% CI)** | **P** | **C** |
| Septal LGE | 1.95 (1.38, 2.76) | <0.001 |  |
| Age (per 10 years) | 1.37 (1.21, 1.55) | <0.00001 | 0.75 |
| NYHA II | 1.19 (0.78, 1.81) | <0.001 |
| NYHA III / IV | 2.33 (1.44, 3.77) |
| RVEF (per 10%) | 0.83 (0.71, 0.96) | 0.011 |
| LVEDVi (per 10) | 1.05 (1.01, 1.10) | 0.023 |
| Male | 1.52 (1.00, 2.32) | 0.049 |
| LVMi (per 10) | 0.96 (0.90, 1.03) | 0.31 |
| LAVi (per 10) | 1.02 (0.96, 1.09) | 0.49 |
| LVEF (per 10%) | 0.96 (0.78, 1.18) | 0.70 |

Each multivariable model is presented (1) without LGE included in the model and (2) with the presence of any LGE and (3) the presence of septal LGE.

**Online Table 4. Sensitivity analysis confirming the association between all-cause mortality and late gadolinium enhancement**

|  |  | **Pts** | **Mortality** | **Adjusted for LVEF, RVEF, NYHA, LVEDVi, LV Mass index, LAVi, Age & Sex** | | | |
| --- | --- | --- | --- | --- | --- | --- | --- |
|  |  | **n** | **n (%)** | **HR (95% CI)** | **P value** | **C statistic** | **AIC** |
| **A**: LGE (Binary) [Any] | 0% | 574 | 73 (12.7) | 1.00 | 0.002 | 0.74 | 1728.6 |
|  | >0% | 300 | 77 (25.7) | 1.70 (1.21, 2.39) |  |  |  |
| **B**: LGE (Binary) [Best] | <1.29% | 617 | 81 (13.1) | 1.00 | 0.002 | 0.74 | 1728.3 |
|  | ≥1.29% | 257 | 69 (26.8) | 1.73 (1.23, 2.43) |  |  |  |
| **C**: LGE (4 Groups) | 0% | 574 | 73 (12.7) | 1.00 | 0.004 | 0.74 | 1729.5 |
|  | >0% & <2.55% | 100 | 24 (24.0) | 1.56 (0.95, 2.58) |  |  |  |
|  | ≥2.55% & <5.10% | 100 | 22 (22.0) | 1.36 (0.83, 2.24) |  |  |  |
|  | ≥5.10% | 100 | 31 (31.0) | 2.20 (1.42, 3.41) |  |  |  |
|  |  |  |  |  |  |  |  |
| **D**: LGE (by Location) | Absent | 574 | 73 (12.7) | 1.00 | 0.002 | 0.75 | 1726.8 |
|  | Septal Only | 42 | 41 (28.9) | 1.81 (1.20, 2.72) |  |  |  |
|  | Free-wall Only | 142 | 4 (9.5) | 0.66 (0.24, 1.81) |  |  |  |
|  | Both | 116 | 32 (27.6) | 2.02 (1.30, 3.14) |  |  |  |
| **E: LGE (Septal)** | **No** | **616** | **77 (12.5)** | **1.00** | **<0.001** | **0.75** | **1723.7** |
|  | **Yes** | **258** | **73 (28.3)** | **1.95 (1.38, 2.76)** |  |  |  |
| **F:** LGE (by Pattern) | Absent | 574 | 73 (12.7) | 1.00 | 0.022 | 0.74 | 1732.8 |
|  | Mid-wall | 25 | 47 (25.4) | 1.59 (1.08, 2.35) |  |  |  |
|  | Sub-EpiCardial | 185 | 4 (16.0) | 1.22 (0.44, 3.40) |  |  |  |
|  | Focal | 68 | 7 (31.8) | 2.33 (1.05, 5.16) |  |  |  |
|  | Multiple | 22 | 19 (27.9) | 1.99 (1.18, 3.36) |  |  |  |

Additional sensitivity analysis adjusting for LVEF, RVEF, NYHA, LVEDVi, LV mass index, LAVi, age & sex confirming the association between all-cause mortality and (A) the presence of late gadolinium enhancement (LGE); (B) the extent of LGE with the largest c-statistic for all-cause mortality; (C) the extent of LGE (as per 3 categories); (D) the location of LGE (septal only, free-wall only or both locations); (E) the presence of septal LGE; (F) the pattern of LGE (linear mid-wall, sub-epicardial, focal or multiple). P values are quoted for each model overall. The model with the smallest Akaike information criterion and the most optimal for prediction of all-cause mortality was (E).

(AIC – Akaike information criterion, CI – confidence intervals, HR – hazard ratio, LGE – late gadolinium enhancement)

**Online Table 5. Univariable and Multivariable models for SCD and aborted SCD**

| **Univariable Models (SCD/ASCD)** | | |
| --- | --- | --- |
|  | **HR (95% CI)** | **P** |
| LGE (binary) | 4.12 (2.64, 6.45) | <0.0001 |
| LVEDVi (per 10%) | 1.09 (1.05, 1.13) | <0.001 |
| LAVi (per 10ml/m^2^) | 1.11 (1.04, 1.18) | <0.001 |
| LV mass index (per 10g/m^2^) | 1.10 (1.03, 1.17) | 0.003 |
| LVEF (per 10%) | 0.79 (0.67, 0.93) | 0.005 |
| RVEF (per 10%) | 0.84 (0.73, 0.97) | 0.016 |
| Male | 1.36 (0.84, 2.20) | 0.22 |
| Age (per 10 years) | 0.91 (0.79, 1.06) | 0.23 |
| NYHA II | 0.76 (0.47, 1.24) | 0.51 |
| NYHA III / IV | 0.99 (0.56, 1.75) |

Multivariable Model 1

| **Multivariable Model 1 without LGE (SCD/ASCD)** | | | |
| --- | --- | --- | --- |
|  | HR (95% CI) | P | C-statistic |
| LVEF | 0.79 (0.68, 0.93) | 0.004 | 0.59 |
| Age (per 10 yrs) | 0.90 (0.78, 1.04) | 0.16 |
| Male | 1.28 (0.79, 2.07) | 0.32 |

| **Multivariable Model 1 with LGE (SCD/ASCD)** | | | |
| --- | --- | --- | --- |
|  | HR (95% CI) | P | C-statistic |
| **Any LGE** | **3.96 (2.41, 6.52)** | **<0.00001** | 0.70 |
| LVEF | 0.88 (0.75, 1.01) | 0.077 |
| Age (per 10 yrs) | 0.89 (0.75, 1.06) | 0.20 |
| Male | 1.01 (0.61, 1.67) | 0.98 |

| **Multivariable Model 1 with LGE** **(by location)** (SCD/ASCD) | | | |
| --- | --- | --- | --- |
|  | HR (95% CI) | P | C-statistic |
| **LGE - Septal Only** | 3.13 (1.68, 5.81) | <0.00001 | 0.72 |
| **LGE- Non-Septal Only** | 2.19 (0.76, 6.31) |
| **LGE - Both** | 5.82 (3.30, 10.27) |
| LVEF | 0.88 (0.76, 1.02) | 0.092 |
| Age | 0.91 (0.76, 1.10) | 0.33 |
| Sex | 1.00 (0.60, 1.67) | 0.99 |

Multivariable Model 2

| **Multivariable Model 2 without LGE (SCD/ASCD)** | | | |
| --- | --- | --- | --- |
|  | HR (95% CI) | P | C-statistic |
| LVEDVi (per 10) | 1.07 (1.01, 1.13) | 0.024 | 0.63 |
| NYHA II | 0.64 (0.38, 1.06) | 0.19 |
| NYHA III / IV | 0.61 (0.30, 1.27) |
| Age (per 10 years) | 0.93 (0.80, 1.08) | 0.32 |
| LAVi (per 10) | 1.05 (0.97, 1.14) | 0.19 |
| Male | 1.03 (0.61, 1.77) | 0.90 |
| RVEF (per 10%) | 0.98 (0.82, 1.17) | 0.82 |
| LVEF (per 10%) | 0.90 (0.70, 1.16) | 0.40 |
| LVMi (per 10) | 0.99 (0.90, 1.09) | 0.87 |

| **Multivariable Model 2 with LGE (SCD/ASCD)** | | | |
| --- | --- | --- | --- |
|  | HR (95% CI) | P | C-statistic |
| **Any LGE** | **3.99 (2.37, 6.69)** | **<0.00001** | 0.72 |
| LVEDVi (per 10) | 1.06 (0.99, 1.13) | 0.076 |
| NYHA II | 0.57 (0.34, 0.98) | 0.079 |
| NYHA III / IV | 0.50 (0.24, 1.06) |
| Age (per 10 years) | 0.92 (0.78, 1.07) | 0.27 |
| LAVi (per 10) | 1.04 (0.96, 1.13) | 0.31 |
| Male | 0.75 (0.42, 1.35) | 0.33 |
| RVEF (per 10%) | 0.96 (0.80, 1.15) | 0.64 |
| LVEF (per 10%) | 0.98 (0.75, 1.28) | 0.88 |
| LVMi (per 10) | 1.00 (0.90, 1.12) | 0.98 |

| **Multivariable Model 2 with LGE (by location) (SCD/ASCD)** | | | |
| --- | --- | --- | --- |
|  | **HR (95% CI)** | **P** | **C** |
| **LGE - Septal Only** | **3.02 (1.55, 5.85)** | <0.00001 | 0.73 |
| **LGE- Non-Septal Only** | **2.17 (0.73, 6.41)** |
| **LGE - Both** | **6.06 (3.44, 10.67)** |
| LVEDVi (per 10) | 1.07 (1.00, 1.14) | 0.034 |
| NYHA II | 0.59 (0.34, 1.01) | 0.12 |
| NYHA III / IV | 0.55 (0.26, 1.17) |
| Age (per 10 years) | 0.92 (0.79, 1.08) | 0.31 |
| Male | 0.76 (0.42, 1.36) | 0.35 |
| LAVi (per 10) | 1.04 (0.96, 1.12) | 0.37 |
| RVEF (per 10%) | 0.94 (0.78, 1.13) | 0.49 |
| LVEF (per 10%) | 1.03 (0.78, 1.36) | 0.86 |
| LVMi (per 10) | 0.99 (0.89, 1.11) | 0.87 |

Each multivariable model is presented (1) without LGE included in the model and (2) with the presence of any LGE and (3) the presence of LGE depending on location.

**Online Table 6. Sensitivity analysis confirming the association between sudden cardiac death events and late gadolinium enhancement**

|  |  | **Pts** | **ASCD & SCD** |  | **Adjusted for LVEF, RVEF, NYHA, LVEDVi, LV Mass index, LAVi, Age & Sex** | | | |
| --- | --- | --- | --- | --- | --- | --- | --- | --- |
|  |  | **n** | **Yes** | **HR (95% CI)** | | **P value** | **C statistic** | **AIC** |
| **A:** LGE (Binary) [Any] | 0% | 574 | 29 (5.1) | 1.00 | | <0.0001 | 0.72 | 1022.8 |
|  | >0% | 300 | 55 (18.3) | 3.99 (2.37, 6.69) | |  |  |  |
| **B**: LGE (Binary) [Best] | <0.71% | 617 | 30 (5.2) | 1.00 | | <0.0001 | 0.72 | 1023.5 |
|  | ≥0.71% | 257 | 54 (18.6) | 3.90 (2.35, 6.49) | |  |  |  |
| **C**: LGE (4 Groups) | 0% | 574 | 29 (5.1) | 1.00 | | <0.0001 | 0.73 | 1023.5 |
|  | >0% & <2.55% | 100 | 13 (13.4) | 2.83 (1.41, 5.66) | |  |  |  |
|  | ≥2.55% & <5.10% | 100 | 18 (18.2) | 3.94 (2.02, 7.69) | |  |  |  |
|  | ≥5.10% | 100 | 24 (23.1) | 5.26 (2.87, 9.64) | |  |  |  |
|  |  |  |  |  | |  |  |  |
| **D: LGE (by Location)** | **Absent** | 574 | **29 (5.1)** | **1.00** | | **<0.0001** | **0.73** | **1018.9** |
|  | **Septal Only** | 42 | **21 (14.8)** | **3.02 (1.55, 5.85)** | |  |  |  |
|  | **Free-wall Only** | 142 | **4 (9.5)** | **2.17 (0.73, 6.41)** | |  |  |  |
|  | **Both** | 116 | **30 (25.9)** | **6.06 (3.44, 10.67)** | |  |  |  |
| **E**: LGE (Septal) | No | **616** | 33 (5.4) | 1.00 | | <0.0001 | 0.72 | 1022.2 |
|  | Yes | **258** | 51 (19.8) | 4.12 (2.44, 6.95) | |  |  |  |
| **F**: LGE (by Pattern) | Absent | 574 | 29 (5.1) | 1.00 | | <0.0001 | 0.73 | 1026.0 |
|  | Mid-wall | 25 | 29 (15.7) | 3.28 (1.82, 5.93) | |  |  |  |
|  | Sub-EpiCardial | 185 | 5 (20.0) | 5.72 (2.11, 15.53) | |  |  |  |
|  | Focal | 68 | 3 (13.6) | 3.61 (0.99, 13.22) | |  |  |  |
|  | Multiple | 22 | 18 (26.5) | 5.21 (2.74, 9.88) | |  |  |  |

Additional sensitivity analysis adjusting for LVEF, RVEF, NYHA, LVEDVi, LV mass index, LAVi, age & sex, confirming the association between all-cause mortality and (A) the presence of late gadolinium enhancement (LGE); (B) the extent of LGE with the largest c-statistic for all-cause mortality; (C) the extent of LGE (as per 3 tertiles); (D) the location of LGE (septal only, free-wall only or both locations); (E) the presence of septal LGE; (F) the pattern of LGE (linear mid-wall, sub-epicardial, focal or multiple). P values are quoted for each model overall. The model with the smallest Akaike information criterion and the most optimal for prediction of all-cause mortality was (E).

(AIC – Akaike information criterion, CI – confidence intervals, HR – hazard ratio, LGE – late gadolinium enhancement)

**Online Table 7A. Late gadolinium enhancement characteristics based on contrast agent administered**

|  | **Gadopentetate dimeglumine (n=303)** | **Gadobutrol**  **(n=571)** | ***P**** |
| --- | --- | --- | --- |
| LGE | 93 (30.7) | 207 (36.3) | 0.12 |
| LGE (%) | 1.80 (4.65) | 2.03 (4.71) | 0.084 |
| LGE Pattern |  |  |  |
| Mid-wall | 57 (18.8) | 128 (22.4) | 0.61 |
| Sub-Epicardial | 7 (2.3) | 18 (3.2) |
| Focal | 7 (2.3) | 15 (2.6) |
| Multiple | 22 (7.3) | 46 (8.1) |
| LGE Location |  |  |  |
| Septal Only | 43 (14.2) | 99 (17.3) | 0.43 |
| Free-wall Only | 14 (4.6) | 28 (4.9) |
| Both | 36 (11.9) | 80 (14.0) |

**Online Table 7**B. Association between late gadolinium enhancement and all-cause mortality based on contrast agent used

|  |  | **Gadopentetate dimeglumine** | | | **Gadobutrol** | | | **Interaction P** |
| --- | --- | --- | --- | --- | --- | --- | --- | --- |
|  |  | **Mortality n (%)** | **HR (95% CI)** | **P** | **Mortality**  **n (%)** | **HR (95% CI)** | **P** |  |
| A: LGE (Binary) [Any] | 0% | 39 (18.6) | 1.00 | - | 34 (9.3) | 1.00 | - | 0.94 |
|  | >0% | 36 (38.7) | 2.42 (1.54, 3.81) | <0.001 | 41 (19.8) | 2.37 (1.50, 3.73) | <0.001 |  |
| B: LGE (Binary) [Best] | <1.29% | 46 (20.3) | 1.00 | - | 35 (9.0) | 1.00 | - | 0.54 |
|  | ≥1.29% | 29 (38.2) | 2.28 (1.43, 3.64) | <0.001 | 40 (22.1) | 2.79 (1.77, 4.39) | <0.0001 |  |
| C: LGE (4 Groups) | 0% | 39 (18.6) | 1.00 | - | 34 (9.3) | 1.00 | - | 0.92 |
|  | >0% & <2.5% | 13 (38.2) | 2.20 (1.17, 4.12) | 0.001 | 9 (14.3) | 1.77 (0.85, 3.70) | 0.002 |  |
|  | ≥2.5% & <5% | 8 (30.8) | 2.12 (0.99, 4.54) |  | 16 (21.9) | 2.57 (1.42, 4.66) |  |  |
|  | ≥5% | 15 (45.5) | 2.90 (1.60, 5.26) |  | 16 (22.5) | 2.66 (1.47, 4.82) |  |  |
|  |  |  |  |  |  |  |  |  |
| D: LGE (by Location) | Absent | 39 (18.6) | 1.00 | - | 34 (9.3) | 1.00 | - | 0.54 |
|  | Septal Only | 16 (17.2) | 2.54 (1.42, 4.56) | <0.0001 | 25 (25.3) | 2.94 (1.76, 4.93) | <0.001 |  |
|  | Free-wall Only | 2 (14.3) | 0.61 (0.15, 2.54) |  | 2 (7.1) | 0.95 (0.23, 3.95) |  |  |
|  | Both | 18 (50.0) | 3.41 (1.95, 5.97) |  | 14 (17.5) | 2.09 (1.12, 3.89) |  |  |
| E: LGE (Septal) | No | 41 (18.3) | 1.00 | - | 36 (9.2) | 1.00 | **-** | 0.62 |
|  | Yes | 34 (43.0) | 3.03 (1.92, 4.78) | <0.0001 | 39 (21.8) | 2.57 (1.64, 4.05) | <0.0001 |  |
| F: LGE (by Pattern) | Absent | 39 (18.6) | 1.00 | - | 34 (9.3) | 1.00 | - | 0.69 |
|  | Mid-wall | 20 (35.1) | 2.25 (1.31, 3.86) | 0.002 | 27 (21.1) | 2.39 (1.44, 3.97) | 0.002 |  |
|  | Sub-Epicardial | 3 (42.9) | 2.59 (0.80, 8.38) |  | 1 (5.6) | 0.72 (0.10, 5.26) |  |  |
|  | Focal | 4 (57.1) | 4.74 (1.69, 13.27) |  | 3 (20.0) | 2.50 (0.77, 8.16) |  |  |
|  | Multiple | 9 (40.9) | 2.28 (1.10, 4.70) |  | 10 (21.7) | 2.90 (1.43, 5.88) |  |  |
